# Policies to Optimize Physician Billing Data in Academic Alternative Relationship Payment Plans: Practices and Perspectives

**DOI:** 10.23889/ijpds.v1i1.383

**Published:** 2017-04-19

**Authors:** Ceara Tess Cunningham, Hude Quan, Nathalie Jette, Tom Noseworthy, Carolyn Decoster

**Affiliations:** 1 Alberta Health Services; 2 University of Calgary

## Objectives

Changes in physician reimbursement policies may hinder the collection of billing claims in administrative databases. Various provincial academic alternative payment programs (APPs) use incentive- or punitive-based tools to motivate physicians to submit billing claims called shadow billings; however, these incentives are not well documented in the literature. We conducted a nation-wide survey and semi-structured face-to-face interviews in Alberta, Canada to determine existing policies and guidelines for incentivizing and promoting physician billing practices.

## Approach

Mail and online surveys were sent out to academic department head physicians in the following provinces: British Columbia, Alberta, Saskatchewan, Manitoba, Ontario, New Brunswick, Prince Edward Island and Newfoundland and Labrador. Face-to-face interviews were conducted in the province of Alberta with managers, government stakeholders, and physicians/administrators from academic APPs and Fee-for-Service plans. Face-to-face interviews and responses by mail and email submission were summarized using content analysis grouped by question type.

## Results

In total, there were 46 respondents (15 interviews, 26 mail/online). Content analysis revealed three primary perspectives, grouped at the level of individual physician, academic, and government. Across all of these unique perspectives, three primary themes emerged: 1) governance; 2) accountability; and 3) funding. Within these themes, findings were categorized as either (a) instruments or tools to promote physician billing in AAPPs; (b) enabling factors to support physician billing in AAPPs; and, (c) constraining factors impeding physician billing in AAPPs.

## Conclusions

According to the majority of our respondents, financial disincentives (i.e. income at risk, financial clawbacks) appear to be most effective as a mechanism to motivate physicians within an academic APP to submit their billings. However, key barriers to successful implementation and delivery of academic APPs include a lack of alignment between government stakeholders, academic leadership and APP physician members and differences in the organizational and accountability structures of APP plans between academic facilities. It is necessary in moving forward to achieve commonly defined standards and frameworks between the various APP models across provinces and academic institutions.

